# Editorial for Special Issue “Recent Advances in Trachea, Bronchus and Lung Cancer Management”

**DOI:** 10.3390/cancers17132147

**Published:** 2025-06-26

**Authors:** Serafeim Chrysovalantis Kotoulas, Dionysios Spyratos, Athanasia Pataka

**Affiliations:** 1 Adult ICU, General Hospital of Thessaloniki “Ippokrateio”, Konstantinoupoleos 49, 54642 Thessaloniki, Greece; 2 Pulmonary Department, Unit of thoracic Malignancies Research, General Hospital of Thessaloniki “G. Papanikolaou”, Aristotle’s University of Thessaloniki, Leoforos Papanikolaou Municipality of Chortiatis, 57010 Thessaloniki, Greece; diospyrato@yahoo.gr; 3 Respiratory Failure Clinic, and Sleep Laboratory, General Hospital of Thessaloniki “G. Papanikolaou”, Aristotle’s University of Thessaloniki, Leoforos Papanikolaou Municipality of Chortiatis, 57010 Thessaloniki, Greece; patakath@yahoo.gr

According to the WHO, deaths from trachea, bronchus, and lung cancers rose to 1.9 million worldwide in 2021, making them the leading cause of cancer-related mortality and the sixth most common cause of death overall. In upper-middle- and high-income countries, however, they ranked fifth [1].

Despite this burden, significant breakthroughs in their management are made each year. The purpose of this Special Issue was to consolidate the most recent advances in the management of trachea, bronchus, and lung cancers—from initial clinical suspicion through diagnosis and treatment options—providing a comprehensive and up-to-date resource to assist clinicians in combatting this lethal disease.

The initial impetus for most randomized clinical trials typically comes from real-world data, which not only supports pharmacovigilance but also enhances our understanding of disease progression, aids in the formulation of external control groups, and addresses critical evidence gaps. For this reason, the European Organization for Research and Treatment of Cancer (EORTC) is developing strategies to generate high-quality real-world evidence by prioritizing pragmatic clinical trials [2]. Such trials have revolutionized lung cancer screening, most notably with the adoption of low-dose computed tomography (LDCT) for at-risk populations [3,4].

Biomarkers represent the next logical step in improving lung cancer screening. They aid in risk stratification for high-risk individuals and help clarify indeterminate pulmonary nodules, reducing overdiagnosis [5]. Once risk is established and suspicious thoracic lesions are detected, obtaining valid histopathological or cytological specimens becomes essential for confirming or excluding malignancy. Conventional bronchoscopy has been widely used for this purpose due to its low complication rates. However, its inability to access distal airways limits its utility compared with transthoracic needle biopsy (TTNB). To address this, robotic bronchoscopy has emerged as a promising new technique with lower complication rates, although the current evidence suggests that it still has a lower diagnostic yield than TTNB, indicating room for further improvement [6].

Identifying not just the type but also the subtype of thoracic malignancies is critical, as different tumors exhibit varying susceptibilities to treatment. For example, distinguishing between lung neuroendocrine tumors (LNETs) and gastroenteropancreatic neuroendocrine tumors (GEP-NETs) is important [7]. Although LNETs represent less than 2% of all lung tumors [8], accurate subtyping is equally important for more common lung cancers to facilitate targeted therapies and personalized treatment. Notable examples include rare mutations in the epidermal growth factor receptor (EGFR), found in 10–15% of lung cancer cases [9], and the Kirsten rat sarcoma virus (KRAS), which has historically been considered undruggable. However, the discovery of the switch II pocket in the KRAS^G12C^ mutant protein has led to the development of direct KRAS^G12C^ inhibitors, changing the therapeutic landscape [10].

Immunotherapy has also emerged in the past decade as a major therapeutic advancement. Immune checkpoint inhibitors (ICIs) are being used as monotherapy, in combination regimens, or alongside chemotherapy in adjuvant, neoadjuvant, and, more recently, perioperative settings. These strategies have significantly influenced the management of early-stage lung cancer and hold promise for broader applications [11]. Although ICIs are also used in patients with disease progression or relapse, their effectiveness is limited, with a median progression-free survival (PFS) of 1.6–3.1 months and a disease control rate (DCR) of 21.4–41.6% [12]. Still, a subset of patients benefit from these therapies, and identifying this population is a critical ongoing challenge [12].

Despite limitations, targeted therapies and ICIs have improved overall prognosis. In a 20-year cohort study from the Institut Curie in France, the median overall survival for metastatic lung cancer increased from 11.1 months (2000–2010) to 15.5 months following the introduction of targeted therapies in 2010, and to 16.2 months after ICIs were introduced in 2018 [13].

Traditional treatments such as radiotherapy have also evolved. Particle therapy, for instance, offers a promising alternative to conventional photon therapy for non-small cell lung cancer (NSCLC), although the heterogeneous structure of lung tissue poses challenges such as Bragg peak degradation and an uneven dose distribution [14].

Lung cancer patients often suffer from comorbidities that can worsen outcomes. Venous thromboembolic disease (VTE), encompassing deep vein thrombosis (DVT) and pulmonary embolism (PE), is a well-known malignancy-related complication that was first identified in the 19th century [15]. Recent evidence supports intermediate-dose Tinzaparin for thromboprophylaxis in high-risk patients as a safe and effective preventive measure [16].

There is also increasing awareness of the interplay between lung cancer and interstitial lung disease (ILD), which share risk factors such as smoking, environmental exposures, and genetic predispositions. These shared pathways impact disease progression and management. As a result, new diagnostic tools, biomarkers, and personalized treatment strategies are essential to improving the outcomes in this vulnerable population [17].

Socioeconomic inequality remains another key factor influencing lung cancer incidence, diagnosis, treatment access, and outcomes. Individuals with lower education levels—often associated with unhealthy behaviors, hazardous occupations, and environmental exposure—face a higher risk of developing lung cancer [18]. Bridging this gap may be possible with the use of artificial intelligence (AI).

AI is poised to play a transformative role in lung cancer management [19]. In screening, AI systems can detect suspicious nodules across various imaging modalities, including chest X-rays, CT scans, and PET scans, with its performance often being comparable to or better than that of experienced radiologists [20,21]. A recent meta-analysis reported a pooled sensitivity and specificity of 0.87, underscoring AI’s diagnostic potential despite study heterogeneity [22]. Additionally, AI can help identify biomarkers years before clinical disease onset, assist in histological and genetic tumor classification, and guide the development of personalized treatment strategies [20,21].

In conclusion, this Special Issue has explored a wide array of topics relating to lung cancer—from diagnostics, biomarkers, and histopathology to comorbidities, novel treatments, real-world studies, and AI applications. As lung cancer remains a rapidly evolving field, continued research is essential and strongly encouraged [23].
